# Effects of enzyme supplementation on growth performance, digestibility of phosphorus, femur parameters and fecal microbiota in growing pigs fed different types of diets

**DOI:** 10.3389/fvets.2024.1413920

**Published:** 2024-06-20

**Authors:** Yi Yin, Maamer Jlali, Bing Yu, Yuheng Luo, Jun He, Ping Zheng, Xiangbing Mao, Hui Yan, Aimin Wu, Shiping Bai, Estelle Devillard, Jie Yu

**Affiliations:** ^1^Key Laboratory of Animal Disease-Resistance Nutrition, Ministry of Education of China, Animal Nutrition Institute, Sichuan Agricultural University, Chengdu, China; ^2^Center of Expertise in Research and Nutrition, Adisseo France S.A.S., Malicorne, France

**Keywords:** phytase, multi-carbohydrase and phytase complex, pigs, mineralization, microbiota

## Abstract

A 42-days study was conducted to evaluate the effects of different dietary types (corn-or wheat-soybean meal-based diet) and phytase (Phy) or a multi-carbohydrase and phytase complex (MCPC) supplementation on growth performance, digestibility of phosphorus (P), intestinal transporter gene expression, plasma indexes, bone parameters, and fecal microbiota in growing pigs. Seventy-two barrows (average initial body weight of 24.70 ± 0.09 kg) with a 2 × 3 factorial arrangement of treatments and main effects of diet type (corn-or wheat-soybean meal-based-diets) and enzyme supplementation (without, with Phy or with MCPC). Each group was designed with 6 replicate pens. The MCPC increased (*p* < 0.05) average daily gain (ADG) and final body weight (BW). A significant interaction (*p* = 0.01) was observed between diet type and enzyme supplementation on apparent total tract digestibility (ATTD) of P. The ATTD of P was higher (*p* < 0.05) in wheat soybean meal-based diets compared to corn-soybean meal-based diets. Compared with the corn-soybean meal-based diet, the relative expression of SLC34A2 and VDR genes in the ileum and SLC34A3 in jejunum of growing pigs fed the wheat-soybean meal based diet was lower (*p* < 0.05). The MCPC significantly reduced (*p* < 0.05) the relative expression of TRPV5 and CALB1 genes in the ileum and increased the expression of CALB1 in the duodenum compared to control diet. The phytase increased (*p* < 0.05) the relative expression of SLC34A1 gene in the duodenum in comparison to control diet and MCPC-supplemented diet. The Ca and P contents in plasma from pigs fed corn-soybean meal-based diet were higher (*p* < 0.05) than those from pigs fed wheat-soybean meal-based diet, and the parathyroid hormone (PTH) and calcitonin (CT) concentrations were lower (*p* < 0.05) than those fed wheat-soybean meal-based diet. The content of Ca and P in the femur and the bone strength of pigs in the corn-soybean meal group were significantly higher (*p* < 0.05) than those in the wheat-soybean meal groups. The phytase increased (*p* < 0.05) the Ca and P content and bone strength of the femur. Additionally, diet type and both enzymes significantly improved fecal microbial diversity and composition. Taken together, diet type and exogenous enzymes supplementation could differently influence the growth performance, utilization of phosphorus, intestinal transporter gene expression, bone mineralization and microbial diversity and composition in growing pigs.

## 1 Introduction

Corn and wheat are often used as energy feed sources for monogastric animals. However, these feed ingredients contain non-starch polysaccharides (NSP), including arabinoxylan, glucan, cellulose and mannans, which can reduce feed efficiency and nutrient digestibility (1). Arabinoxylan is the most common NSP found in cereals grains such as wheat and corn which can exert an antinutrient effect in monogastric animals since they do not have endogenous enzymes able to digest arabinoxylan (2). Hence, a multi-carbohydrase containing xylanase, beta-glucanase and arabinofuranosidase in the diets can produce oligosaccharides from arabinoxylan degradation by random hydrolysis of β-1,4-glycosidic bonds (1). Additionally, in these plant-based feed ingredients, up to 80% of the total phosphorus is in the form of phytate generally known as an antinutrient (3, 4). Due to the negligeable endogenous enzyme activity within the gut, the phytate could not be degraded in the intestinal tract to improve the availability of P (5). Therefore, phytase is commonly used in the feed for monogastric animals. The supplementation of phytase in swine diets can improve not only the digestibility of calcium and phosphorus but also that of amino acids and energy and reduce the excretion of phosphorus in animal manure (6).

Moreover, phosphorus and calcium are two closely related minerals in animals because they are regulated by the same hormones, including vitamin D, parathyroid hormone, fibroblast growth factor 23 and calcitonin (7–9). These hormones play key roles in the absorption, accumulation, resorption, and excretion pathways of P and Ca (10). In addition to hormones, we assumed that diet type and enzyme supplementation with phytase alone or in combination with a multi-carbohydrase can influence the gene expression related to Ca and P absorption along the intestinal tract of growing pigs.

Compared with corn-based diets, previous study reported that in pigs, the wheat-based diets can significantly change the composition of microbiota and the concentration of microbial metabolites in the gut probably due that wheat-based diets contain more NSP than diets based on corn which may affect the hindgut microbes (11). Some other authors have found that the addition of xylanase in the diet significantly affects the abundance of microorganisms in the intestinal tract of pigs (12). Thus, the effects of phytase and enzyme mixture supplementation in the corn and wheat-based diets on growth performance, bone mineralization and gut microbiota in growing pigs remained elusive. Therefore, this study aimed to investigate the effects of different dietary types (corn- or wheat-soybean meal-based diet) and phytase (Phy) or a multi-carbohydrase and phytase complex (MCPC) supplementation on growth performance, digestibility of P, intestinal transporter gene expression, plasma indexes, bone parameters and fecal microbiota composition and diversity in growing pigs.

## 2 Materials and methods

### 2.1 Experimental design and diets

Seventy-two healthy DLY [Duroc × (Landrace × Yorkshire)] barrows (70-d of age) with body weight (BW) of 24.70 ± 0.09 kg were used in a 42-d trial after a 5-d adaptation. Barrows were assigned into 6 treatment groups in a randomized complete block design involving a 2 diet types × 3 enzymes supplementation factorial arrangement of treatments using body weight as the blocking factor. Two diet types include corn-soybean meal-based diet or wheat-soybean meal-based diet. Enzyme supplementation includes without any supplementation, with a 6-phytase expressed in *Buttiauxella* spp., Phy or with a multi-carbohydrase and phytase complex (MCPC) which is a mixture of xylanase, *β*-glucanase and *α*-arabinofuranosidase and a 6-phytase at optimal dose (Rovabio Advance Phy, Adisseo France SAS). At day 36 of the experimental period, 0.5% chromium dioxide was added to the feed as an indigestible marker for digestibility determination. Each treatment consisted of 6 replicate pens with 2 pigs per pen. The ambient temperature is controlled at 26 ± 2°C, and the relative humidity is controlled at 60% ± 5%.

Two types of diets were formulated according to the recommended nutrient requirements for growing pigs (NRC 2012). Experimental diets included a corn-soybean meal-based diet or a wheat-soybean meal-based diet. The phytase was added to provide at least 1,000 FTU/kg diet, and the MCPC was supplemented to supply at least 1,800 U of xylanase, 1,244 U of β-glucanase, and 1,000 FTU of phytase/kg diet. The Composition and calculated nutrient levels of the experimental diets were presented in Table 1. All diets were antibiotics-free and mashed. Experimental diets and water were offered *ad libitum* throughout the experimental period.

**Table 1:** Diet composition and analyzed nutrient content of the experimental diets (as-fed basis).

	Corn-soybean meal			Wheat-soybean meal		
	Without	Phy	MCPC	Without	Phy	MCPC
Ingredient, %						
Corn	67.73	67.73	67.73	–	–	–
Wheat	–	–	–	81.00	81.00	81.00
Soybean meal 48%	25.33	25.33	25.33	11.83	11.83	11.83
Soybean oil	3.00	3.00	3.00	3.09	3.09	3.09
Chloride choline	0.10	0.10	0.10	0.10	0.10	0.10
Monocalcium phosphate	0.90	0.90	0.90	0.45	0.45	0.45
Limestone	1.40	1.40	1.40	1.38	1.38	1.38
NaCl	0.30	0.30	0.30	0.30	0.30	0.30
MCPC	–	–	0.01	–	–	0.01
l-lysine HCl 95%	0.53	0.53	0.53	0.84	0.84	0.84
DL-methionine 98.5%	0.04	0.04	0.04	0.05	0.05	0.05
l-threonine 98.5%	0.07	0.07	0.07	0.20	0.20	0.20
l-isoleucine 99%	–	–	–	0.07	0.07	0.07
l-valine 98%	–	–	–	0.09	0.09	0.09
Premix^1^	0.10	0.10	0.10	0.10	0.10	0.10
Chromium oxide	0.50	0.50	0.50	0.50	0.50	0.50
Nutrient contents^2^, %						
NE, kcal/kg	2,531	2,531	2,531	2,534	2,534	2,534
Crude protein	17.01	16.96	17.03	17.00	16.88	16.91
Ca	0.71	0.72	0.71	0.73	0.75	0.74
Total P	0.55	0.55	0.56	0.49	0.49	0.53
Digestible P	0.30	0.30	0.30	0.30	0.30	0.30
SID lysine	1.26	1.26	1.26	1.26	1.26	1.26
SID Met + Cys	0.61	0.61	0.61	0.61	0.61	0.61
SID threonine	0.70	0.70	0.70	0.71	0.71	0.71
SID tryptophan	0.19	0.19	0.19	0.19	0.19	0.19
SID isoleucine	0.67	0.67	0.67	0.68	0.68	0.68
SID valine	0.79	0.79	0.79	0.79	0.79	0.79

^1^Premix, per kilogram of diet, 8,800 IU of vitamin A, 880 IU of vitamin D, 64 IU of vitamin E, 4 mg of vitamin K (menadione sodium bisulfite), 70 μg of vitamin B12, 14 mg of riboflavin, 60 mg of d-pantothenic acid, 30 mg of niacin, 6 mg of vitamin B6, 200 μg of biotin, 1.2 mg of folic acid, 120 mg of Fe (as iron carbonate), 25 mg of Mn (as manganese oxide), 17 mg of Cu (as copper chloride), 0.3 mg of I (as ethylenediamine dihydroiodide), 0.2 mg of Se (as sodium selenite), and 120 mg of Zn (as zinc oxide). ^2^The values of crude protein, Ca and total P were actually measured, the others were calculated. Phy: phytase; MCPC: a multi-carbohydrase and phytase complex.

### 2.2 Sample collection

On day 39 of the experimental period, fecal samples were collected from each replicate pen and during 4 consecutive days, at least 100 g of fresh fecal samples in each pen were collected with disposable gloves during defecation to avoid falling to the ground and put them into the sample bag immediately, then 10% of dilute sulfuric acid and 4–5 drops of toluene for nitrogen fixation and preservation of the fresh stool in a 1:10 ratio (13). After mixing, put it in −20°C for refrigeration in time. On day 43 and after 12 h of fasting, 6 pigs per treatment (1 pig per pen) were selected according to the average weight for blood Research Topic. Blood samples were centrifuged at 3,500 × *g* for 10 min at 4°C. Then, plasma samples were stored at −20°C for further analysis. After blood sampling, all pigs were euthanized. Then the abdominal cavity of the pig was opened, and the duodenum, jejunum, and ileum were quickly separated according to the anatomical structure. The duodenum, ileum, and approximately 10 cm jejunum (the same part for each pig) were cut longitudinally, and gently rinsed with 0.9% pre-cooled normal saline. Then, a sterile microscope slide was used to gently scrape the intestinal mucosa into a sterile frozen storage tube (each segment of the intestine requires a new fragment, and the entire operation is operated on ice), and then stored it in −80°C to facilitate the further determination. At the end of experiment, rectal digesta were collected with sterile swab and tubes directly from the anus before stored at −80°C. Femur samples were collected and weighed, then quickly stored at −20°C for subsequent analysis.

### 2.3 Growth performance

After fasting for 12 h, pigs in each pen were weighed at the beginning of the study and at 42d using platform scale. Feed intake was measured by recording the added and remained feed in the trough, and the average daily gain (ADG) and feed-to-gain (F:G) ratio based on the feed intake were calculated.

### 2.4 Measurements in plasma samples

The content of calcium and phosphorus in plasma and the activity of alkaline phosphatase (ALP) were determined using commercial kits (CAT# A059-1-1, Nanjing Jiancheng Bioengineering Institute, Jiangsu, China); vitamin D3 (CAT# 1593), parathyroid hormone (PTH, CAT# 12481), and calcitonin (CT, CAT# 5225) contents were determined by spectrophotometer (Pharmacia, Cambridge, United Kingdom) with the corresponding enzyme-linked immunosorbent assay kits (Jiangsu Meimian Industrial, Inc., Nanjing, China).

### 2.5 Diets, feces and femurs analyses

The femurs were analyzed for bone breaking strength at Wuhan Pinjian Testing Technology Co., Ltd. (Wuhan, China). Then, the cortical cross-sectional diameter (internal and external) was measured with a digital caliper, and the geometric characteristics of the femur were determined according to previously reported method (14). Fecal samples were dried in an oven at 65°C, then diets and fecal samples were crushed and passed through a 0.5 mm screen. Femur samples were dried at 105°C for 24 h to determine the dry matter. The ash content was determined by burning samples in a muffle furnace 550°C for 24 h followed the method 942.05, AOAC, 2006. The calcium and phosphorus was analyzed by a spectrophotometer (Pharmacia, Cambridge, United Kingdom) after washing at 600°C followed method 965.17; AOAC, 2006 (P) and method 968.08; AOAC, 2006 (Ca).

### 2.6 Relative quantitative real-time PCR

The frozen small intestinal mucosa sample (about 0.1 ~ 0.2 g) was crushed into powder (liquid nitrogen was constantly added to keep the temperature low during the grinding process), and then added to a sterile centrifuge tube containing 1 mL RNAiso Plus reagent (Takara, Dalian, China), and centrifuged at 12,000 *g* for 15 min at 4°C (ThermoMicro17R, Thermo Fisher Scientific Inc., Waltham, United States). Then, the total RNA was extracted from mucosa samples collected from the duodenum, jejunum, and ileum. For each sample, a spectrophotometer (NanoDrop Technologies, Inc., Wilmington, DE, United States) was used to verify the concentration and quality of total RNA at 260 nm and 280 nm. The optical density ratio (260 nm/280 nm) was between 1.8 and 2.0. The integrity of RNA was checked by formaldehyde agarose gel electrophoresis. Reverse transcription of each sample was performed using the Prime Script RNART kit (Dakara Biotechnology Company, Dalian, China).

QuantStudio 5 real-time PCR detection system and SYBR reagent (Takara, Dalian, China) were used to quantitatively detect SLC34A1(Na^+^-Pi cotransporter 1); SLC34A2 (Na^+^-Pi cotransporter 2); SLC34A3(Na^+^ -Pi cotransporter 3); TRPV5 (transient receptor potential vanilloid 5); TRPV6 (transient receptor potential vanilloid 6); CALB1 (calbindin); PMCA1b (plasma membrane Ca^2+^ adenosintriphosphatase); VDR (vitamin D receptor); and FGF23 (fibroblast growth factor 23) mRNA expression levels using GAPDH as a housekeeping. These specific primers were synthesized commercially and purchased from Sanguang Biotechnology Co., Ltd. (Shanghai, China) and were listed in Supplementary Table S1. The 10 μL qRT-PCR system consists of 5 μL SYBR (Dalian Takara), 0.5 μL forward primer, 0.5 μL reverse primer, 3 μL ribozyme H_2_O, and 1 μL cDNA template. The reaction was carried out at 95°c for the 30s, denatured at 95°c for 40 times for 5 s, annealing for 30s, and finally extended at 72°c for 5 min. Through melting curve analysis, the correctness of PCR amplification was confirmed. Use the 2^−∆∆Ct^ method to calculate the relative expression rate of the target gene relative to the reference gene (15).

### 2.7 Fecal 16S rRNA

The fecal microbial flora structure was analyzed by 16S rRNA amplicon sequencing, and the V3–V4 region of the bacterial 16S rRNA hypervariable region was sequenced and analyzed. First, fecal microbial DNA was extracted using the QIAamp DNA Stool Mini Kit (Qiagen, GmbH Hilden, Germany) according to the provided manual, and 1% agarose gel electrophoresis was used to detect the quality of DNA, and 2% agarose gel electrophoresis was used to detect the size of PCR product bands. All this process was completed by Beijing Nuohe Zhiyuan Biological Company (Beijing, China). Briefly, the raw data were preprocessed to eliminate adapter pollution and low quality to obtain clean reads. The paired-end clean reads with overlaps were merged to tags by Connecting Overlapped Pair-End (COPE) software. Bacterial tags were clustered into operational taxonomic units (OTUs) based on 97% sequence similarity by scripts of UCHIME software. Sequence analysis was performed by Uparse software (Uparse v7.0. 1,001). Sequences with ≥97% similarity were assigned to the same OTUs. Then, the Silva Database was used based on the Mothur algorithm to annotate taxonomic information for each representative sequence. Subsequent analyses of alpha diversity and beta diversity were performed based on this output normalized data. PCoA (principal coordinates analysis) analysis was performed using the WGCNA package, stat packages and ggplot2 package in R software (Version 2.15.3). A heatmap was visualized using R software, and log 10 transformation was applied to the bacterial relative abundance data matrix (16).

### 2.8 Statistical analysis

The PROC MIXED procedure of SAS 9.4 (SAS Institute, Inc., Cary, NC, United States) was used to analyze all data except the growth performance in the trial. The model included the fixed effects of diet type (corn-soybean meal diet and wheat-soybean meal diet), enzyme effect (without enzyme, Phy addition, and MCPC addition), and their interaction, and the random effect of block. For the data of the growth performance, the above model additionally included the initial body weight as covariant. The pen was used as the experimental unit. Statistical significance was declared at *p* < 0.05, and 0.05 ≤ *p* < 0.10 was considered as statistical trend.

## 3 Results

### 3.1 Growth performance

The effect of diet type and enzyme supplementation on growth performance of growing pigs is shown in Table 2. No significant interaction was observed between diet type and enzyme supplementation on all performance parameters. No significant difference between pigs fed corn- or wheat-soybean meal-based diet on growth performance. Compared with pigs fed the diets without enzyme supplementation, phytase addition tended (*p* = 0.08) to improve final BW (+4.8%) and ADG (+8.2%), while MCPC addition improved (*p* = 0.02) final BW and ADG by 6.3 and 10.6%, respectively.

**Table 2:** Effects of diet type and enzyme supplementation on growth performance of growing pigs.

Item	Diet type (DT)		Enzyme (Enz)			SEM^1^	*p*-value		
	Corn-SBM	Wheat-SBM	Without	Phy	MCPC		BD	Enz	BD × Enz
Initial BW^2^, kg	24.70	24.71	24.70	24.70	24.71	0.09	0.95	0.99	0.99
Final BW, kg	62.97	62.86	60.66^b^	63.58^ab^	64.50^a^	1.23	0.85	0.02	0.23
ADG^3^, kg/pig/d	0.91	0.91	0.86^b^	0.93^ab^	0.95^a^	0.03	0.84	0.02	0.23
ADFI^4^, kg/pig/d	1.92	1.88	1.82	1.91	1.96	0.06	0.41	0.12	0.35
*F*:*G*^5^	2.11	2.07	2.13	2.07	2.07	0.04	0.23	0.24	0.82

Means (*n* = 6 replicate pen per treatment) with different superscripts (a, b) in the same row differ (*P* < 0.05). ^1^SEM, standard error of the mean. ^2^BW, body weight. ^3^ADG, average daily body weight gain. ^4^ADFI, average daily feed intake. ^5^*F*:*G*, the ratio of average daily feed intake: average daily gain. Phy, phytase; MCPC, a multi-carbohydrase and phytase complex.

### 3.2 Digestibility of phosphorus

**Figure 1:**
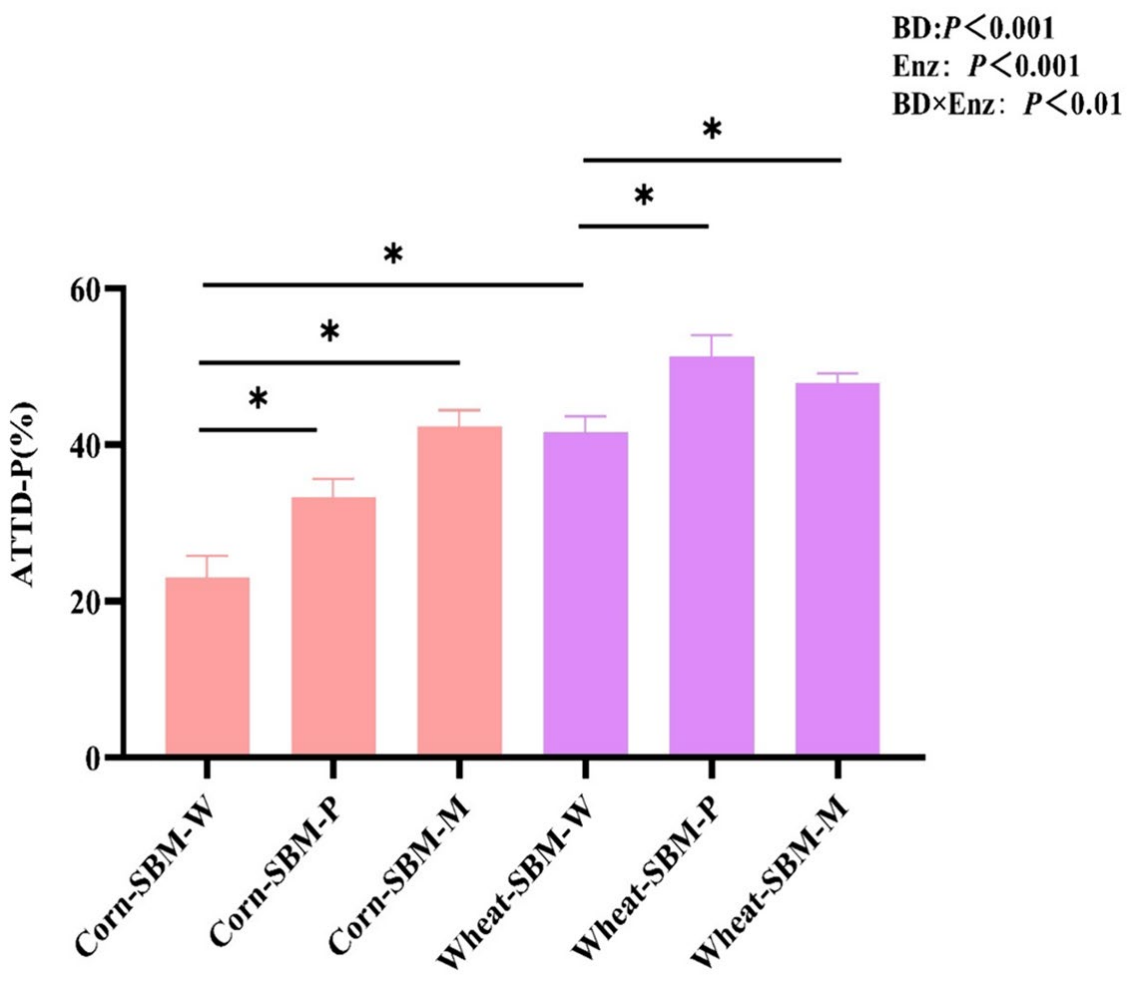
Effects of diet type and enzyme supplementation on the apparent total tract digestibility of phosphorus (ATTD of P) in growing pigs. Values are the means (*n* = 6 replicates per treatment). ATTD-P, apparent total tract digestibility of *P*. Statistical significance was assessed at *p < 0.05.

As shown in Figure 1, a significant interaction (*p* = 0.01) was observed between diet type and enzyme supplementation on ATTD of P. Compared to pigs fed the corn-soybean meal diet without enzymes, the ATTD of P was improved (*p* < 0.05) by 10.3 and 19.5% points with Phy and MCPC, respectively. In comparison to pigs fed the wheat-soybean meal diet without enzymes, the addition of phytase and MCPC improved the ATTD of P by 9.8 and 6.4% points, respectively.

### 3.3 Intestinal gene expression

The effects of diet type and enzyme supplementation on the relative expression of genes related to calcium and phosphorus absorption in the duodenum, jejunum and ileum of growing pigs are presented in Table 3. Significant interactions (*p* < 0.05) were observed between diet type and enzyme supplementation on the expression of *TRPV6* and *VDR* in the ileum and *FGF23* in the duodenum. Compared with the corn-soybean meal-based diet, the relative expression of *SLC34A2* and *VDR* genes in the ileum and *SLC34A3* in jejunum of growing pigs fed the wheat-soybean meal-based diet was lower (Table 3, *p* < 0.05). The addition of MCPC in the diet significantly reduced (*p* < 0.05) the relative expression of *TRPV5* and *CALB1* genes in the ileum and increased the expression of *CALB1* in the duodenum compared to control diet. The addition of phytase in the diet increased (*p* < 0.05) the relative expression of *SLC34A1* gene in the duodenum in comparison to control diet and MCPC-supplemented diet.

**Table 3:** Effects of diet type and enzyme supplementation on gene expressions of intestinal calcium and phosphorus absorption in growing pigs.

Item^1^	Gut site	Diet type (DT)		Enzyme (Enz)			SEM^2^	*P*-value		
		Corn	Wheat	Without	Phy	MCPC		BD	Enz	BD × Enz
SLC34A1	Duodenum	2.86	3.97	2.97^ab^	5.66^a^	1.62^b^	1.11	0.23	0.01	0.72
	Jejunum	1.85	1.55	1.58	2.28	1.23	4.58	0.51	0.18	0.80
	Ileum	1.17	2.04	1.59	2.38	0.85	16.53	0.14	0.13	0.11
SLC34A2	Duodenum	2.03	1.87	2.10	2.23	1.51	0.84	0.86	0.65	0.50
	Jejunum	1.25	1.38	1.19	1.03	1.72	2.13	0.87	0.41	0.07
	Ileum	0.75^A^	0.38^B^	0.72	0.57	0.39	0.46	0.002	0.08	0.38
SLC34A3	Duodenum	1.02	2.89	1.92	1.31	2.63	1.18	0.06	0.57	0.68
	Jejunum	0.98^A^	0.68^B^	0.87^ab^	0.55^b^	1.07^a^	0.30	0.05	0.04	0.56
	Ileum	0.96	0.77	0.89	0.94	0.77	0.68	0.43	0.80	0.40
TRPV5	Duodenum	1.65	1.60	1.76	2.10	1.02	0.66	0.91	0.30	0.38
	Jejunum	1.55	1.57	1.25	2.10	1.32	1.23	0.95	0.41	0.86
	Ileum	0.97	0.98	1.58^a^	0.86^ab^	0.47^b^	0.41	0.99	0.02	0.62
TRPV6	Duodenum	1.29	1.65	1.57	1.77	1.07	0.37	0.26	0.19	0.33
	Jejunum	2.15	1.83	1.76	2.30	1.91	1.43	0.55	0.77	0.22
	Ileum	0.90	3.76	5.21^a^	1.12^b^	0.67^b^	0.79	<0.001	<0.001	<0.001
CALB1	Duodenum	1.09	1.00	1.06	0.81	1.26	0.21	0.57	0.13	0.27
	Jejunum	1.29	1.53	1.59	1.73	0.92	0.65	0.54	0.32	0.25
	Ileum	1.02	0.91	1.41^a^	1.03^a^	0.45^b^	0.32	0.55	<0.001	0.06
PMCA1b	Duodenum	0.96	1.08	0.99^ab^	0.78^b^	1.28^a^	0.15	0.34	0.01	0.14
	Jejunum	0.96	1.13	1.14	0.96	1.03	0.25	0.33	0.68	0.75
	Ileum	1.19	1.11	1.12	1.37	0.95	0.23	0.65	0.16	0.42
VDR	Duodenum	1.07	0.97	1.05	0.96	1.04	0.13	0.39	0.84	0.26
	Jejunum	1.09	0.86	1.10	1.00	0.82	0.33	0.21	0.42	0.90
	Ileum	1.26	0.85	1.19	1.21	0.77	0.20	0.02	0.06	0.05
FGF23	Duodenum	2.12	1.91	1.11^b^	2.91^a^	2.03^ab^	0.64	0.61	0.03	0.01
	Jejunum	1.58	1.49	1.40	2.08	1.12	2.21	0.84	0.26	0.90
	Ileum	1.61	1.45	2.07	1.62	0.89	14.21	0.93	0.58	0.19

Means (*n* = 6 replicates per treatment) with different superscripts (a, b) in the same row differ (*P* < 0.05). ^1^SLC34A1, Na^+^-Pi cotransporter 1, SLC34A2, Na^+^-Pi cotransporter 2, SLC34A3, Na + -Pi cotransporter 3, TRPV5, transient receptor potential vanilloid 5, TRPV6, transient receptor potential vanilloid 6, CALB1, calbindin, PMCA1b, plasma membrane Ca^2+^ adenosintriphosphatase, VDR, vitamin D receptor, FGF23, fibroblast growth factor 23. ^2^SEM, standard error of the mean. Phy, phytase; MCPC, a multi-carbohydrase and phytase complex.

### 3.4 Plasma indexes

There was a significant interaction (*p* < 0.05) between diet type and enzyme supplementation on plasma P content and ALP activity (Table 4). Pigs fed wheat-soybean meal-based diet without enzyme supplementation or supplemented with MCPC exhibited lower (*p* < 0.05) plasma P content compared with pigs fed corn without enzyme supplementation. Additionally, plasma ALP activity was lower (*p* < 0.05) in pigs fed corn-soybean meal diet without enzyme supplementation or wheat-soybean meal-diet supplemented with phytase compared with pigs fed corn- or wheat-supplemented with MCPC. Compared with pigs fed corn-soybean meal-based diet, pigs fed wheat-soybean meal-based diet presented lower (*p* < 0.05) plasma Ca content and higher (*p* < 0.05) PTH and CT. Compared with pigs fed diets without enzyme addition, plasma Ca content decreased (*p* = 0.01) by MCPC supplementation, while it was not influenced by phytase supplementation.

**Table 4:** Effects of diet type and enzyme supplementation on plasma indexes in growing pigs.

Item^1^	Diet type (DT)		Enzyme (Enz)			SEM^2^	*P*-value		
	Corn-SBM	Wheat-SBM	Without	Phy	MCPC		BD	Enz	BD × Enz
Ca, μmol/mL	0.72^A^	0.56^B^	0.75^a^	0.63^ab^	0.53^b^	0.07	0.01	0.02	0.51
P, mmol/L	3.12	2.68	2.82	3.04	2.84	0.12	<0.001	0.15	0.05
ALP, U/L	19.00	19.09	15.38	16.33	25.42	2.39	0.79	<0.001	0.04
PTH, ng/L	22.45^B^	24.42^A^	22.59	23.73	23.99	0.79	0.006	0.20	0.70
1,25(OH)_2_D, ng/L	29.83	31.07	31.28	30.11	29.97	1.14	0.24	0.55	0.09
CT, ng/L	20.24^B^	22.66^A^	21.54	21.26	21.54	0.79	0.001	0.92	0.94

Means (*n* = 6 replicates per treatment) with different superscripts (A, B or a, b) in the same row differ (*P* < 0.05). ^1^ALP, alkaline phosphatase; PTH, parathyroid hormone; CT, calcitonin. ^2^SEM, standard error of variance. Phy, phytase; MCPC, a multi-carbohydrase and phytase complex.

### 3.5 Femur mineralization

There were significant interactions (*p* < 0.05) between diet type and enzyme supplementation on femur weight, femur P and Ca contents (Table 5). Compared with pigs fed the corn-soybean meal-based diet, the bone strength, the contents of Ca and P in the femurs were significantly reduced (*p* < 0.05) in growing pigs fed the wheat-soybean meal-based diet. The addition of MCPC to the diet significantly increased (*p* < 0.05) the length of the femur. Supplementation of both enzymes to the diet significantly increased (*p* < 0.05) bone strength in the femur. Supplementation of phytase in the diet significantly increased (*p* < 0.05) calcium and phosphorus content in the femur (Table 5). Femur weight was higher in the wheat-soybean meal diet supplemented with MCPC than those in the other treatments.

**Table 5:** Effects of diet type and enzyme supplementation on femur parameters in growing pigs.

Item^1^	Diet type (DT)		Enzyme (Enz)			SEM^2^	*P*-value		
	Corn	Wheat	Without	Phy	MCPC		BD	Enz	BD × Enz
Weight, g	207.32	204.71	200.38	205.51	212.14	5.88	0.60	0.15	0.03
Length, cm	16.80	16.66	16.43^b^	16.68^ab^	17.07^a^	0.20	0.40	0.01	0.10
Bone strength,	3620.49^A^	3019.28^B^	2762.50^b^	3742.07^a^	3455.08^a^	328.46	0.04	0.02	0.32
CSA, mm^2^	232.71	221.65	214.06	234.50	233.00	14.82	0.38	0.33	0.29
MRWT, mm	1.20	1.13	1.16	1.20	1.15	0.08	0.28	0.83	0.16
CI, %	77.35	67.53	66.95	74.77	75.60	6.56	0.09	0.38	0.36
P, % of ash	9.25	8.92	9.20	9.59	8.47	0.14	0.01	<0.001	0.001
Ca, % of ash	18.94	18.16	18.23	19.16	18.26	0.27	0.001	0.003	<0.001

Means (*n* = 6 replicates per treatment) with different superscripts (A, B or a, b) in the same row differ (*P* < 0.05). ^1^CSA, cross-sectional area, MRWT, mean relative wall thickness, CI, cortical index. ^2^SEM, standard error of variance. Phy, phytase; MCPC, multi-carbohydrase and phytase complex.

### 3.6 Fecal microbiota

**Figure 2:**
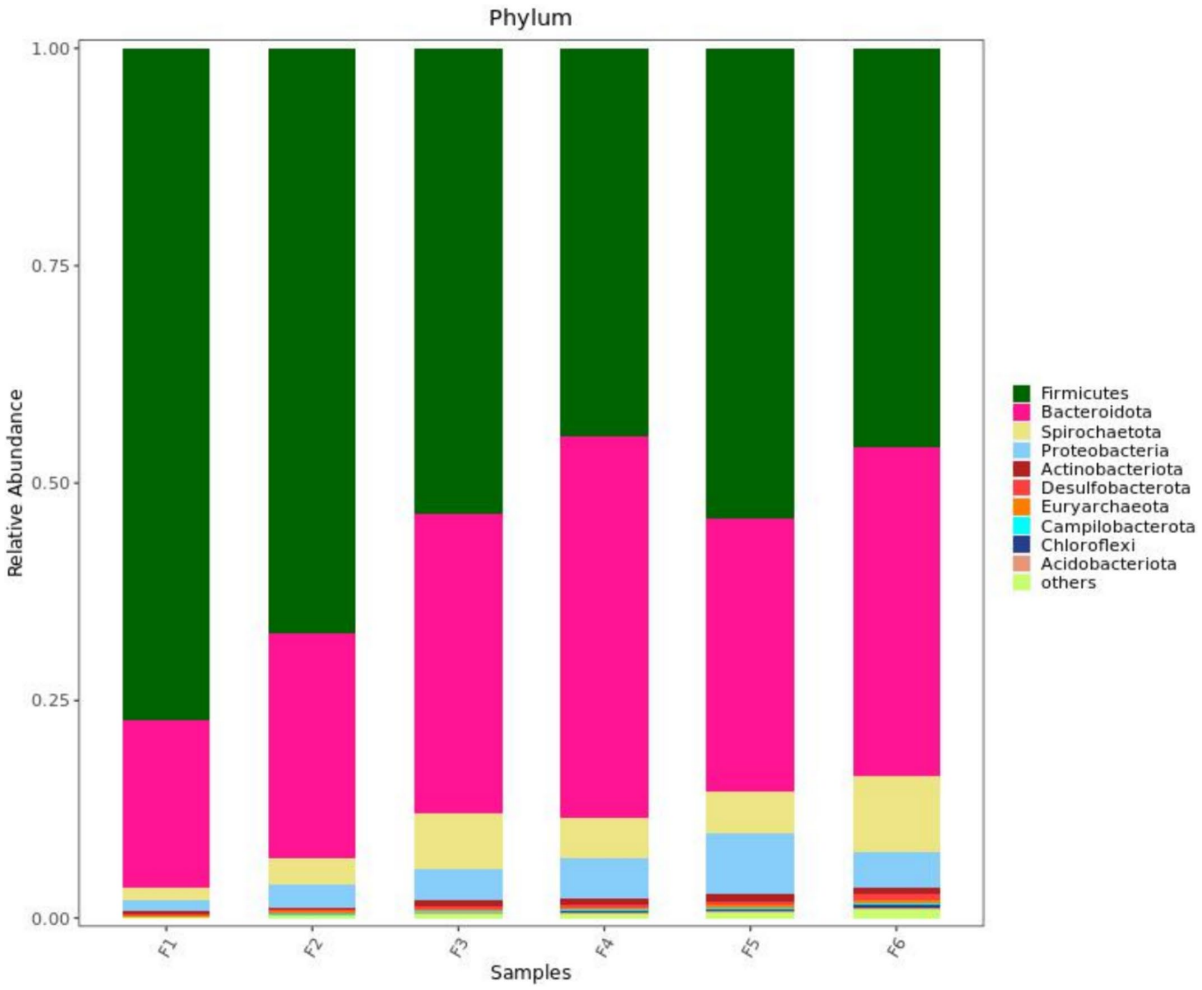
Effects of diet type and enzyme supplementation on the relative abundance of fecal microbial phyla (top 10). F1 = fecal microorganisms of corn-soybean meal group, F2 = fecal microorganisms of corn-soybean meal + phytase group, F3 = fecal microorganisms in corn-soybean meal + MCPC group, F4 = fecal microorganisms of wheat-soybean meal group, F5 = fecal microorganisms of wheat-soybean meal + phytase group, F6 = Fecal microorganisms of wheat- soybean meal + MCPC group.

Figure 2 shows the fecal microorganisms (top 10 phyla levels) in growing pigs fed different types of diets with or without enzyme supplementation, we can find Firmicutes and Bacteroidota predominant. After adding enzyme in corn-soybean meal group, the abundance of Bacteroidota in feces increased and the abundance of Firmicutes decreased, but this phenomenon was not observed after adding enzyme in wheat-soybean meal group. Table 6 shows the fecal microbial alpha diversity of growing pigs fed different types of diets (with or without enzyme preparations). Compared with the corn-soybean meal-based diet, the Shannon and Simpson indices of fecal microorganisms of growing pigs fed the wheat-soybean meal-based diet was increased (*p* < 0.05), and the diversity of fecal microorganisms was significantly increased (*p* < 0.05). Also adding both enzymes to the diet significantly increased the fecal microbial Shannon and Simpson indices. Fecal microbial Shannon and Simpson indices were higher in the MCPC-fed group than in the other treatments.

**Table 6:** Effects of diet type and enzyme supplementation on fecal microbial alpha diversity in growing pigs.

Item	Diet type (DT)		Enzyme (Enz)			SEM^1^	*P*-value		
	Corn	Wheat	Without	Phy	MCPC		BD	Enz	BD × Enz
Shannon	7.85	8.72	7.87	8.25	8.74	0.121	<0.001	<0.001	<0.001
Simpson	0.97	0.99	0.97	0.99	0.99	0.004	<0.001	<0.001	<0.001

**Figure 3:**
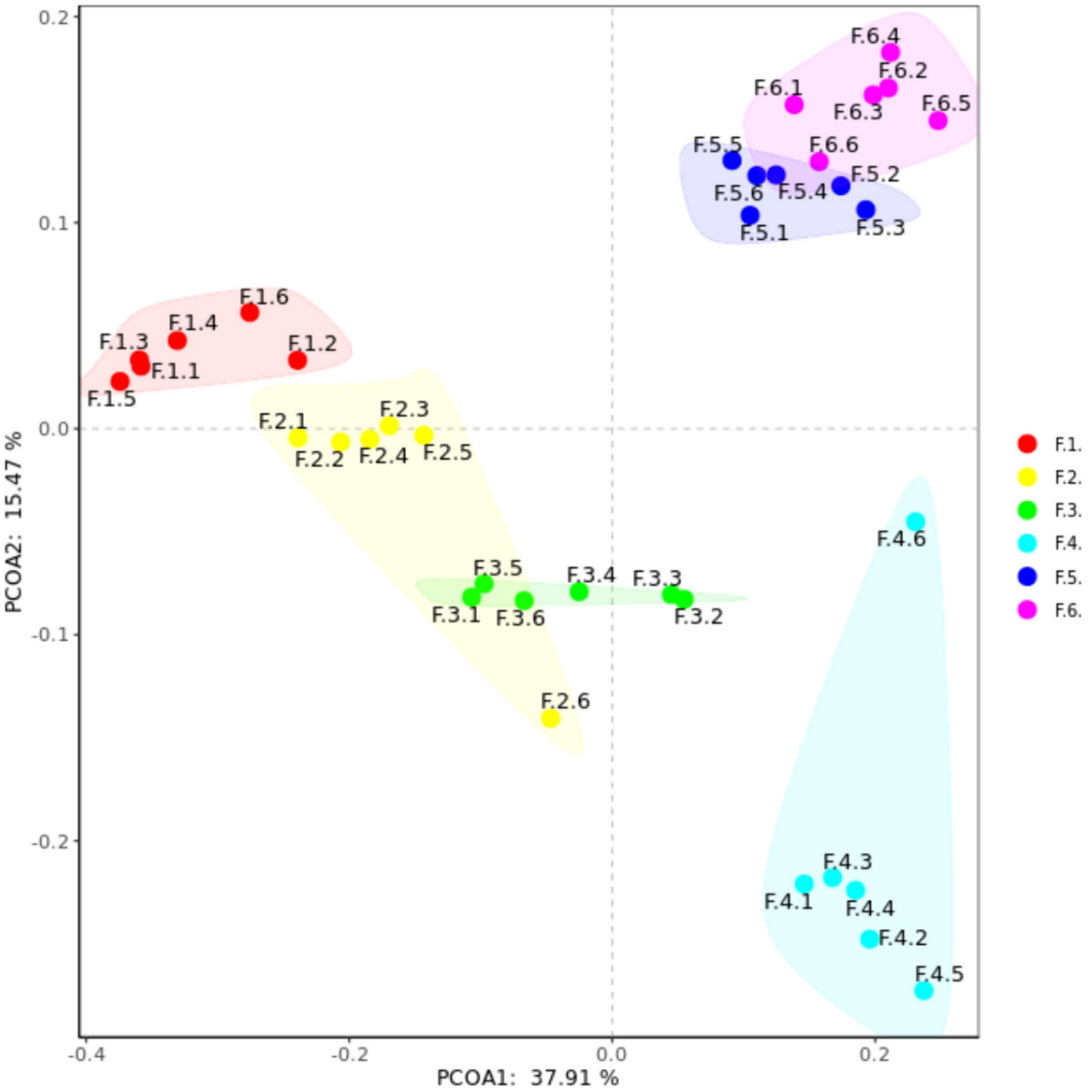
Principal coordinate analysis (PCoA) composition of fecal microorganisms in each group. F1 = fecal microorganisms of corn-soybean meal group, F2 = fecal microorganisms of corn-soybean meal + phytase group, F3 = fecal microorganisms in corn-soybean meal + MCPC group, F4 = fecal microorganisms of wheat-soybean meal group. F5 = fecal microorganisms of wheat-soybean meal + phytase group, F6 = fecal microorganisms in wheat-soybean meal + MCPC group.

**Figure 4:**
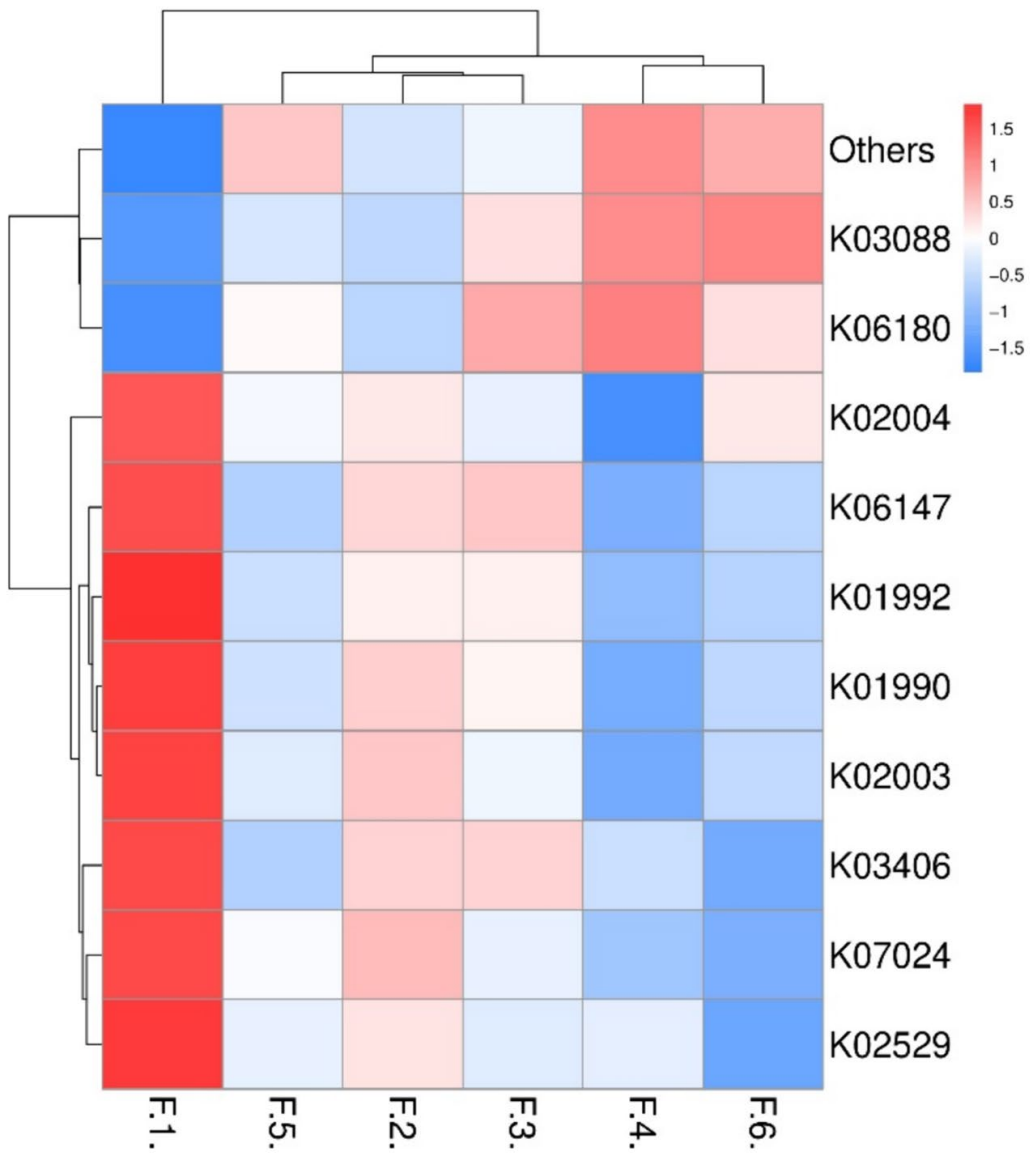
Prediction of fecal microbial function (top 10). F1 = fecal microorganisms of corn-soybean meal group, F2 = fecal microorganisms of corn-soybean meal + phytase group, F3 = fecal microorganisms of corn-soybean meal + MCPC group, F4 = fecal microorganisms of wheat- soybean meal group. F5 = fecal microorganisms of wheat-soybean meal + phytase group, F6 = fecal microorganisms in wheat-soybean meal + MCPC group.

As shown in Figure 3 the fecal microbes are located on the coordinates of each treatment to form a clear regional separation, which indicates that there are differences in the composition of fecal microbes among the treatments. Among them, changing the diet type made each coordinate point farther away, which indicated that the diet type treatment had a greater impact on the composition of fecal microbial structure. Further prediction of fecal microbial functions can reveal that metabolism, through KEGG processing analysis, found that these functions are Two-component system, Starch and sucrose, Biosynthesis of secondary metabolites and Bacterial chemotaxis. As shown in Figure 4, the proportion of fecal microorganisms performing the above functions was more in the corn soybean meal without enzyme group.

## 4 Discussion

It has been widely reported that exogenous phytase and xylanase added alone or in combination in growing pigs’ diets could improve the growth performance (17, 18). In this experiment, phytase supplementation tended to increase ADG and final BW, while the addition of MCPC, enriched in xylanase, *β*-glucanase, and phytase significantly increased final BW and ADG compared with pigs fed the control diet. This result is similar with previous studies that observed that supplementation of multi-carbohydrase and phytase in combination improved the growth performance in pigs probably by targeting the two main antinutritional factors namely the NSP and phytate (17, 18). Previous studies reported that phytase and NSP-degrading enzymes could hydrolyze phytate and NSP which lead to increase the availability of nutrients for pigs and consequently the growth performance (17, 18).

In the present study, the addition of phytase or MCPC increased the ATTD of P, resulting in an increase in the availability of digestible P in the diets which can improve the growth performance of growing pigs. This result is in agree with the previous study (19). Moreover, calcium and phosphorus are actively absorbed in the porcine small intestine mainly through transporter-mediated transcellular pathways. In the present study, the expression levels and signaling of calcium-phosphorus carrier proteins in the gut may represent the actual retention of calcium and phosphorus in the gut. Coupled with the mediation of vitamin D signaling, it can be used for further transport and absorption of calcium and phosphorus in the gut. The SLC34 family of sodium-driven phosphate cotransporters is comprised of three members: NaPi-IIa (SLC34A1), NaPi-IIb (SLC34A2), and NaPi-IIc (SLC34A3) (20). From the gene expression levels in this experiment, the main active carrier proteins mediating calcium and phosphorus transport were highly expressed in the duodenum and jejunum. Compared with the corn-soybean meal-based diet, feeding the wheat-soybean meal-based diet could significantly reduce the expression level of *SLC34A2* gene in the ileum, but feeding the wheat-soybean meal-based diet significantly increased ATTD of P in growing pigs. While reducing the dietary digestible phosphorus level, previous study found that the gene expression of Na+-PiIIb transporter was significantly decreased, but the protein expression level of this protein was significantly increased (21). The Na+-PiIIb transporter is a member of the SLC family (22). This suggests that more *SLC34A3* mRNA translated to functional protein, and improve the digestibility of phosphorus (22). The addition of MCPC to the diet significantly increased the expression level of the *SLC34A3* gene in the jejunum, which was consistent with the changes in our ATTD of P. During calcium-saturated transcellular processes, calcium is taken up by enterocytes through calcium channels such as *TRPV6* located in the brush border membrane. In this experiment, the relative expression of *TRPV6* gene in the ileum of growing pigs was significantly increased by feeding the wheat-soybean meal-based diet, indicating that calcium absorption was enhanced in this treatment group. The relative expression of the ileal *TRPV6* gene was significantly reduced by the addition of both enzymes in the diet.

Compared with the corn-soybean meal-based diet, feeding the wheat-soybean meal-based diet significantly reduced plasma calcium and phosphorus levels in growing pigs. While increasing the intestinal absorption of calcium, there will be also a feedback regulation system to maintain the level of calcium in the plasma (22). The CT content in the plasma of the wheat-soybean meal group was significantly increased, which could also maintain the calcium concentration in the plasma at a certain level. Secondly, the digestion and absorption of phosphorus is also affected by many factors. After changing the type of diet, PTH in the plasma of the wheat-soybean meal group was significantly increased, and PTH could reduce the number of NaPi-IIA and NaPi-IIC proteins on the brush border membrane (BBM) (23, 24), thereby limiting the absorption of part of P, It was finally reflected in the decrease of Ca and P contents in the plasma of the wheat-soybean meal group.

In this study, diet type and enzymes (with and without supplementation) had no significant effect on femur length, cross-sectional area (CSA), cortical index (CI) and mean relative wall thickness (MRWT) general characteristic parameters of growing pigs. Since bone strength is provided by an inorganic component consisting of hydroxyapatite and insoluble salts containing calcium and phosphorus, the content of phosphorus and calcium in the femur increased after dietary supplementation of phytase, with a corresponding increase in bone strength. In the previous studies (25), it was also found that adding phytase significantly increased the content of calcium and phosphorus in the tibia of piglets after the 95-day experiment in piglets. Similarly, after the 185-day experiment in fattening pigs, adding phytase significantly increased fattening. Calcium and phosphorus content were increased in pig metacarpal bones, indicating that the addition of phytase in the diet significantly increased the deposition of calcium and phosphorus in the femur, improved the bone strength, and was beneficial to the bone development of growing pigs. Also, the interaction between diet types and enzymes on P content and femur weight were observed in this study, showing a better effect to add enzymes in corn-soybean meal diet. The addition of phytase in the diet based on barley - wheat - soybean meal also significantly increased the content of calcium and phosphorus in the bone (25). There was also a significant interaction between diet type and enzyme in Ca and P contents in the bones, showing more Ca and P in the bone of pigs fed corn-soybean meal diet or diets with enzyme supplementations.

In this experiment, it is not difficult to see from the results and analysis of 16S sequencing that changing the type of diet and adding phytase or MCPC can significantly affect the alpha diversity of fecal microorganisms, such as the Shannon and Simpson indices are significantly increased. Secondly, we can also see from the Beta analysis that changing the type of diet and adding phytase or MCPC can produce significant differences in the structural composition of fecal microorganisms, but changing the type of diet has a greater impact on the difference in the structural composition of pig feces in terms of microbiota. This may be because wheat contains a large amount of NSP, and NSP is the main component of dietary fiber. They are not digested and absorbed in the small intestine, but are fermented by the resident microbiota in the gut (26). Most gut bacteria preferentially ferment carbohydrates, which also include NSP, implying that diets containing large amounts of complex structural NSP may nourish a greater number and variety of microbes (27).

Taken together, the results of the present study demonstrated that diet type and exogenous enzymes supplementation could differently influence the growth performance, utilization of P, intestinal transporter gene expression, bone mineralization and microbial diversity and composition in growing pigs.

## Data availability statement

The datasets for this study can be available on request to the corresponding author. The raw sequencing data are available from NCBI repository: https://www.ncbi.nlm.nih.gov/, under accession number PRJNA1123310.

## Ethics statement

The animal study was approved by Institutional Animal Care and Use Committee of Sichuan Agricultural University. The study was conducted in accordance with the local legislation and institutional requirements.

## Author contributions

YY: Data curation, Investigation, Visualization, Writing – original draft. MJ: Conceptualization, Data curation, Software, Validation, Visualization, Writing – review & editing. BY: Data curation, Investigation, Methodology, Writing – review & editing. YL: Data curation, Software, Writing – review & editing. JH: Investigation, Writing – review & editing. PZ: Investigation, Writing – review & editing. XM: Investigation, Writing – review & editing. HY: Writing – review & editing. AW: Investigation, Writing – review & editing. SB: Data curation, Software, Writing – original draft. ED: Project administration, Validation, Writing – original draft. JY: Conceptualization, Funding acquisition, Resources, Supervision, Validation, Writing – review & editing.
