# Supplementary material for: Effects of enzyme supplementation on growth performance, digestibility of phosphorus, femur parameters and fecal microbiota in growing pigs fed different types of diets
Source: Front Vet Sci. 2024 Jun 20;11:1413920. doi: 10.3389/fvets.2024.1413920 (PMC11223171; doi:10.3389/fvets.2024.1413920)
Supplement: Supplementary file 1 [file Table_1.DOCX]

**Table S1.** Sequences for [real-time PCR](https://www.sciencedirect.com/topics/agricultural-and-biological-sciences/real-time-polymerase-chain-reaction) primers.^1^

| Genes | Accession No. | Primer sequences (5′→3′) | | Size, bp |
| --- | --- | --- | --- | --- |
| *SLC34A1* | NM_001044623.1 | F | TCAACTCTCTGCTCAAGGGC | 183 |
|  |  | R | CACCTAGGCCAATGAGTGGG |  |
| *SLC34A2* | NM_001256772.1 | F | AACCTCCATCACCAACACCC | 280 |
|  |  | R | AAGAGCACCAACACGGAGAG |  |
| *SLC34A3* | NW_018084833.1 | F | GTACCACAACAGGATGCCGA | 256 |
|  |  | R | AGAGGACCCTGAACCACTGA |  |
| *TRPV5* | XM_021078896.1 | F | TCCCTGTAACTTGCCAGTGC | 103 |
|  |  | R | TGCTGATCCCAGTCTTGCTG |  |
| *TRPV6* | FJ268731.2 | F | GAATGCGGTTGCATTGAGCA | 112 |
|  |  | R | TTACACCCTTTCCACAGCCG |  |
| *CALB1* | NM_001130226.1 | F | ATTTCGACGCTGACGGAAGT | 224 |
|  |  | R | TTGCTGGCATCGGAATAGCA |  |
| *PMCA1b* | NM_214352.3 | F | GAAAATGGTTCCTGCTGCC | 275 |
|  |  | R | GCAACCGAGTTGTTTGCCAT |  |
| *VDR* | NM_001097414.1 | F | TGGTTGGAAGTGTCTGGGAG | 117 |
|  |  | R | GGGGTCAGGTAAGGAAGTGC |  |
| *FGF23* | XM_001926525.4 | F | CAGCTACCACCTGCAGATCC | 146 |
|  |  | R | CCCTTAAGTCCATGCAGAGG |  |
| *GAPDH* | NM_001206359.1 | F | TGAAGGTCGGAGTGAACGGAT | 114 |
|  |  | R | CACTTTGCCAGAGTTAAAAGCA |  |

^1^ Abbreviation: PCR, polymerase chain reaction; *SLC34A1,* Na+-Pi cotransporter; *SLC34A2,* Na+-Pi cotransporter; *SLC34A3*, Na+ -Pi cotransporter; *TRPV5*, transient receptor potential vanilloid; *TRPV6,* transient receptor potential vanilloid; *CALB1,* calbindin; *PMCA1b*, plasma membrane Ca2+ adenosintriphosphatase; *VDR,* vitamin D receptor; *FGF23*, fibroblast growth factor 23; *GAPDH*, glyceraldehyde-3phosphate dehydrogenase
